# Recent Trends and Future Direction of Dental Research in the Digital Era

**DOI:** 10.3390/ijerph17061987

**Published:** 2020-03-18

**Authors:** Tim Joda, Michael M. Bornstein, Ronald E. Jung, Marco Ferrari, Tuomas Waltimo, Nicola U. Zitzmann

**Affiliations:** 1 Department of Reconstructive Dentistry, University Center for Dental Medicine Basel, University of Basel, 4058 Basel, Switzerland; n.zitzmann@unibas.ch; 2 Department of Oral Health & Medicine, University Center for Dental Medicine Basel, University of Basel, 4058 Basel, Switzerland; michael.bornstein@unibas.ch (M.M.B.); tuomas.waltimo@unibas.ch (T.W.); 3 Department of Reconstructive Dentistry, Center for Dental Medicine Basel, University of Zurich, 8032 Zurich, Switzerland; ronald.jung@zzm.uzh.ch; 4 Department of Prosthodontics & Dental Material, University School of Dental Medicine, University of Siena, 53100 Siena, Italy; ferrarm@gmail.com

**Keywords:** digital transformation, rapid prototyping, augmented and virtual reality (AR/VR), artificial intelligence (AI), machine learning (ML), personalized dental medicine, tele-health, patient-centered outcomes

## Abstract

The digital transformation in dental medicine, based on electronic health data information, is recognized as one of the major game-changers of the 21st century to tackle present and upcoming challenges in dental and oral healthcare. This opinion letter focuses on the estimated top five trends and innovations of this new digital era, with potential to decisively influence the direction of dental research: (1) rapid prototyping (RP), (2) augmented and virtual reality (AR/VR), (3) artificial intelligence (AI) and machine learning (ML), (4) personalized (dental) medicine, and (5) tele-healthcare. Digital dentistry requires managing expectations pragmatically and ensuring transparency for all stakeholders: patients, healthcare providers, university and research institutions, the medtech industry, insurance, public media, and state policy. It should not be claimed or implied that digital smart data technologies will replace humans providing dental expertise and the capacity for patient empathy. The dental team that controls digital applications remains the key and will continue to play the central role in treating patients. In this context, the latest trend word is created: augmented intelligence, e.g., the meaningful combination of digital applications paired with human qualities and abilities in order to achieve improved dental and oral healthcare, ensuring quality of life.

## 1. Introduction

Digital transformation is the ubiquitous catchword in a variety of business sectors, and (dental) medicine is no exception [1]. Continuous progress in information technology (IT) has made it possible to overcome the limitations and hurdles that existed in clinical and technological workflows just a few years ago [2]. In addition, social and cultural behaviors of civilized society in industrial countries have changed and fostered the trend of digitalization: urbanism, centralization, and mobility, permanent accessibility via smartphones and tablets combined with the internet of things (IoT), as well as convenience-driven markets striving for efficiency [3].

The implementation of digital tools and applications reveals novel options facing today’s chief problems in healthcare, such as a demographic development of an aging population with an increased prevalence of chronic diseases and increased treatment costs over an individual’s lifespan [4]. In dental medicine, several digital workflows for production processing have already been integrated into treatment protocols, especially in the rapidly growing branch of computer-aided design/computer-aided manufacturing (CAD/CAM) and rapid prototyping (RP) [5].

New possibilities have opened up for automated processing in radiological imaging using artificial intelligence (AI) and machine learning (ML). Moreover, augmented and virtual reality (AR/VR) is the technological basis for the superimposition of diverse imaging files creating virtual dental patients and non-invasive simulations comparing different outcomes prior to any clinical intervention. Increased IT-power has fostered these promising technologies, whose possible uses can only be assessed in the future [6]. Not all digital options are currently exhausted, and their (valuable) advantages are not completely understood. Basic science, clinical trials, and subsequently derived knowledge for innovative therapy protocols need to be re-directed towards patient-centered outcomes, enabling the linkage of oral and general health instead of merely industry-oriented investigations [7].

To sum up, unseen opportunities will arise due to digital transformation in oral healthcare and dental research. Therefore, this opinion letter highlights the estimated top five healthcare trends and innovations of the dawning digital era that might influence the direction of dental research and their stakeholders in the near future.

## 2. Top Five Healthcare Trends and Innovations

### 2.1. Rapid Prototyping (RP)

RP is a technique to quickly and automatically construct three-dimensional (3D) models of a final product or a part of a whole using 3D-printers. The additive manufacturing process allows inexpensive production of complex 3D-geometries from various materials and minimal material wastage [8]. However, while the future looks very promising from a technical and scientific point of view, it is not clear how RP and its products will be regulated. This uncertainty is problematic for the producing industry, healthcare provider, and patients as well.

In dentistry, one of the main difficulties today is the choice of materials. Commercially available materials commonly used for RP are currently permitted for short to medium-term intraoral retention only and are, therefore, limited to temporary restorations and not yet intended for definitive dental reconstructions. RP offers great potential in dental technology for mass production of dental models, but also for the fabrication of implant surgical guides [9]. For those indications, prolonged intraoral retention is not required. From an economic point of view, a great advantage is the production in large quantities at the same time in a reproducible and standardized way. Another important area of application is the use of 3D-printed models in dental education based on CBCT or µCT. An initial study, however, has revealed that 3D-printed dental models can show changes in dimensional accuracy over periods of 4 weeks and longer. In this context, further investigations comparing different 3D-printers and material combinations are compellingly necessary for clarification [10].

In the near future, those material-related barriers and limitations will probably be broken down. Many research groups are focusing on the development of printable materials for dental reconstructions, such as zirconium dioxide (ZrO_2_) [11]. This different mode of fabrication of ZrO_2_ structures could allow us to realize totally innovative geometries with hollow bodies that might be used, for example, for time-dependent low-dose release of anti-inflammatory agents in implant dentistry [12]. A completely revolutionary aspect would be the synthesis of biomaterials to artificially create lost tooth structures using RP technology [13]. Instead of using a preformed dental tooth databank, a patient-specific digital dental dataset could be acquired at the time of growth completion and used for future dental reconstructions. Furthermore, the entire tooth can be duplicated to serve as an individualized implant. RP will most likely offer low-cost production and highly customized solutions in various fields of dental medicine that can be tailored to suit the specific needs of each patient.

### 2.2. Augmented and Virtual Reality (AR/VR)

AR is an interactive technology enhancing a real-world environment by computer-animated perceptual information. In other words, AR expands the real world with virtual content. In most cases, it is the superimposition of additional digital information on live images or videos. VR, in contrast, uses only artificial computerized scenarios without connection to reality [14]. Depending on the technique, every conceivable way of sensation can be used, mainly visual, auditory, and haptic, independently or in any combination [15]. Today, there is a rapidly increasing number of applications for AR/VR technologies in dental medicine as a whole, as well as many intriguing developments for both patients and healthcare providers [16,17,18].

AR/VR software allows users to superimpose virtually created visualizations onto recordings of the patient in natural motion. Any 3D-model, for instance, a prosthetic design of a possible reconstruction, can be augmented into the individual patient situation to simulate diverse, prospective outcomes in advance without invasive work steps [19]. These digital models can then be viewed in real-time and facilitate communication not only with the patient to demystify the complex treatment steps but also between dental professionals to make the treatment more predictable and efficient. In the future, the possibilities will continue to grow and help facilitate the dental routine. An interesting indication is the augmentation of CBCT-based virtual implant planning directly into the oral cavity or while using intraoral scanners (IOS), projection, and display of the optically detected area with AR glasses.

Another promising area of interest is the sector of dental education, transferring theoretical knowledge and practical exercises to offer interactive teaching with 24/7-access and objective evaluation. AR/VR-based motor skill training for tooth preparation especially facilitates efficient and autonomous learning for dental students. Initial studies have shown that AR/VR technologies stimulate more senses to learn meritoriously [20]. Moreover, in postgraduate education, challenging and complex clinical protocols can be trained in a complete virtual environment without risk or harm for real patients; additionally, specialists can continuously maintain their skills while training with AR/VR-simulations. Within a few years, AR/VR will have the potential to revolutionize dental education radically [21,22].

### 2.3. Artificial Intelligence (AI) and Machine Learning (ML)

AI (including ML) has already invaded and established itself in our daily lives, although in more subtle means, such as virtual assistants named “Siri” or “Alexa”. The basis for AI is the increasing power of computers to think like and complete tasks currently performed by humans with greater speed, accuracy, and lower resource utilization [23,24]. Therefore, AI technology is perfect for work that requires the analysis and evaluation of large amounts of data. Repetitive activities are boring and tiring for humans in the long-run with increased risk of error, while AI-based applications do not show signs of fatigue. In contrast to humans, the artificial learning process results in constant better performance with increasing workload. Additionally, computers are not biased compared to humans, who come with innate biases and may judge things prematurely and differently from each other [25,26].

The most valuable indication for the use of AI and ML in dentistry is the entire field of diagnostic imaging in dento-maxillofacial radiology [27,28]. Currently, applications and research in AI purposes in dental radiology focus on automated localization of cephalometric landmarks, diagnosis of osteoporosis, classification/segmentation of maxillofacial cysts and/or tumors, and identification of periodontitis/periapical disease. Computer software analyzing radiographs has to be trained on huge datasets (“big data”) to recognize meaningful patterns. The diagnostic performance of AI models varies among different algorithms used, is also dependent on the observers labeling the datasets, and it is still necessary to verify the generalizability and reliability of these models by using adequate, representative images. AI software must be able to understand new information presented by images as well as written text or spoken language with proper context. Finally, the software must be able to make intelligent decisions regarding this new information, and then, learn from mistakes to improve the decision-making for future processing [29].

A beneficial AI system should realize all of this in about the same time that a human being can perform the given task. Up to now, applications of AI on a broad scale were not technically feasible or cost-effective, so the reality of AI has not yet matched the possibilities in routine dental applications [30], although the technical progress is exponential, and very soon, a large number of AI models will be developed for automated diagnostics of 3D-imaging identifying pathologies, prediction of disease risk, to propose potential therapeutic options, and to evaluate prognosis.

### 2.4. Personalized (Dental) Medicine

Electronic health records (eHR) with standardized diagnostics and generally accepted data formats are the mandatory door opener to personalized medicine and predictive models investigating a broader population. The structured assessment and systematic collection of patient information is an effective instrument in health economics [31]. Health data can be obtained from routine dental healthcare and clinical trials, as well as from diverse new sources, as IoT in general, and specifically, data on the social determinants of health [3].

The linkage of individual patient data gathered from various sources enables the diagnosis of rare diseases and completely novel strategies for research [32]. Examining large population-based patient cohorts could detect unidentified correlations of diseases and create prognostic models for new treatment concepts. The linkage of patient-level information to population-based citizen cohorts and biobanks provides the required reference of diagnostic and screening cutoffs that could identify new biomarkers through personalized health research [33].

eHR has great power for a change of research both ways. On the other side, the digitized transparent patient could be stigmatized and categorized by insurance companies, provoking adverse effects that have not yet been determined socially [3,6]. Therefore, linked biomedical data supporting register-based research pose several risks and methodological challenges for clinical research: appropriate security settings and the development of algorithms for statistical calculations, including interpretation of collected health data [34,35]. A generally accepted code of conduct has to be defined and established for the ethical and meaningful use of register-based patient data.

Overall, personalized medicine holds the key to unlocking a new frontier in dental research. Genomic sequencing, combined with the developments in medical imaging and regenerative technology, has redefined personalized medicine using novel molecular tools to perform patient-specific precision healthcare [36,37]. It has the potential to revolutionize healthcare using genomics information for individual biomarker identification [38]. The vision is an interdisciplinary approach to dental patient sample analysis, in which dentists, physicians, and nurses can collaborate to understand the inter-connectivity of disease in a cost-effective way [39].

### 2.5. Tele-Healthcare

Tele-healthcare enables a convenient way for patients to increase self-care while potentially reducing office visits and travel time [40]. Considering the growing number of the elderly population with reduced mobility and/or nursing home-stay, special-care patients, as well as people living in rural areas, these patient groups would benefit significantly from tele-dentistry [41,42]. Measures to be taken in case of dental trauma can be effectively communicated by telephone counselors and can be frequently used during out-of-office hours [43]. In general, it facilitates easier access to care and also represents a cost-reduced option for patients, as instead of expensive treatments, tele-dentistry shifts towards prevention practices and allows patients to consult with otherwise unavailable dental professionals, for example, using a live consult via video-streaming [44,45]. Nevertheless, it must be emphasized that tele-dentistry can never replace a real dentist; rather, it must be understood as an additional tool [30].

Today, tele-dentistry is only in an early start-up phase [46]. Early studies have mainly focused on specific and rare diseases that might require surgery, but there are findings that suggest that a teleradiology system in general dental practice could be helpful for the differential diagnosis of common lesions and may result in a reduction of unnecessary costs [47]. There is a fundamental need to regulate the expanding field of tele-healthcare, with guidelines to secure clinical quality standards. The legislation must be clearly defined and clarified for routine implementation of a national-wide tele-dentistry platform. The technical requirements must be met and security standards for sensitive patient information guaranteed, with well-defined regulatory affairs.

## 3. Conclusions

The future direction of dental research should foster the linkage of oral and general health in order to focus on personalized medicine considering patient-centered outcomes. In this context, dental research must have an impact as a deliverable to society, not just research to churn out scientific publications but to truly change protocols applied in the clinic. Moreover, here, digitization with AI/ML and AR/VR represents the most promising tools for innovative research today. Furthermore, research in a digital era will also be more and more assessed in terms of “impact” as a deliverable good. Impact assessment is still very much debated by scientists, healthcare policy-makers, and politicians. Additionally, general public health societies are increasingly dependent on solid data sets, gaining knowledge to enable innovations and result in recommendations, guidelines, and healthcare policies of utmost importance. These are supposed to generate economic and social benefits on every and each level from an individual to a population. Scientists in dental medicine have also to be aware that funding might be increasingly dependent on the possibility to demonstrate an impact on a large scale. Thus, the use of impact assessments in the future will most likely serve the following two tasks: (1) demonstrating the value of research, and (2) increasing the value of research through a more effective way of financing research in order to have a societal impact [48,49].

For digital dentistry, this requires managing expectations pragmatically and ensuring transparency for all stakeholders: patients, healthcare providers, university and other research institutions, the medtech industry, insurance, public media, and state policy. It should not be claimed or implied that digital smart data technologies will replace humans who possess dental expertise and the capacity for patient empathy. Therefore, the dental team controlling the power of the digital toolbox is the key and will continue to play a central role in the patient’s journey to receive the best possible individual treatment, and to provide emotional support. The collection, storage, and analysis of digitized biomedical patient data pose several challenges. In addition to technical aspects for the handling of huge amounts of data, considering internationally defined standards, an ethical and meaningful policy must ensure the protection of patient data for safety optimal impact.

Nowadays, the mixed term “augmented intelligence” is perhaps somewhat prematurely introduced in social media. However, the benefits of digital applications will complement human qualities and abilities in order to achieve improved and cost-efficient healthcare for patients. Augmented intelligence based on big data will help to reduce the incidence of misdiagnosis and offers more useful insights—quickly, accurately, and easily. This is all achievable without losing the human touch, improving the quality of life.
